# CD71: Role in permafrost immunity

**DOI:** 10.6026/973206300200208

**Published:** 2024-03-31

**Authors:** Francesco Chiappelli

**Affiliations:** 1 Dental Group of Sherman Oaks, Sherman Oaks, CA 91403, USA; 2 UCLA Center for the Health Sciences, Los Angeles, CA 90095, USA

**Keywords:** CD71, permafrost immunity, transferrin receptor, Arenaviruses, CD81

## Abstract

Iron, an essential constituent of cell metabolism, is transported intra-cellularly bound to the ubiquitous 76 kDa blood glycoprotein transferrin via the transferrin receptor, CD71. Because of its structure, CD71 facilitates the binding and penetration of a large variety of viruses into the host. Among which the hemorrhagic fever-causing New World mammarena viruses (family of single stranded ambisense segmented RNA Arenaviridae), the single stranded positive sense RNA hepatitis C virus, the single stranded negative sense segmented influenza A virus, the single stranded negative sense RNA rabies virus, the single stranded positive sense SARS-CoV2 and possibly many others. In this process, CD71 is associated with the target of the anti-proliferative antibody-1 (CD81) viral co-receptor. In light of the plethora of novel and ancient viruses and microbes emerging from melting eternal glacier ice and permafrost, it is timely and critical to define and characterize interventions, besides the soluble form of CD71 (sCD71), that can abrogate or minimize this novice non-canonical function of CD71.

## The transferrin receptor:

Iron is an essential constituent of cell metabolism, but excess iron leads to oxidative stress within the cells. The fine regulation of iron influx into the cell is mediated by its 76 kDa blood transport glycoprotein, transferrin, via the transferrin receptor (TfR). Excess iron is exported from the cell via ferroportin-1 [1,2]. There are two types of transferrin receptors: the high affinity TfR1 is ubiquitously expressed and finely regulated during cell activation and proliferation; TfR2 expression is restricted to certain cell types and is unaffected by intracellular iron concentrations. TfR1, designated as Cluster of Differentiation (CD)71, consists of a type-II transmembrane glycoprotein expressed as a disulfide-linked homo-dimer on the cell surface. Each monomer binds one holo-transferrin molecule creating the iron-transferrin-CD71 complex that is endo-cytosed by the cell through a clathrin-dependent process. The trans-membrane segment of the molecule serves both as a signal for chain translocation and as a membrane anchor [3,4]. The number of CD71 moieties is the rate-limiting step for iron entry into cells and its expression is precisely controlled and regulated, particularly in functionally active and rapidly proliferating cells [5]. CD71 is unregulated during the acute-phase response to infection [6]. Excess CD71 is truncated from the plasma membrane, resulting in the soluble form of TfR1, viz sCD71, a clinical sign of iron deficiency in inflammatory states [7].

## CD71 and the Trojan horse metaphor:

CD71, in part because of its very structure, can and does bind to alternate ligands primarily via binding to its sialic acid residues [3-6]. These ligands are thus internalized into the cell cytoplasm, thus revealing CD71 as a molecular Trojan Horse. To be sure, the Trojan Horse was not a horse: what Homer described in the Odyssey, that is that the Ancient Greeks erected a large wooden horse within which some of their best armed men hid as it was proffered as an offering to the enemy city of Troy, and from which they sprang in the middle of the night to set fire to the city and bring victory to the Greeks, was actually not a horse at all. Recent findings have demonstrated that the contraction was a boat - a type of Phoenician boat called ἵππoς that is hippos, which translates to a horse: a boat whose hull at the bow was so prominent as to resemble the front of a horse (hence the name), and so voluminous as to serve as a large container ship for commerce. The hippos' hull was spacious enough to house temporarily a contingent of armed men. The subterfuge was to bring in the loaded Hippos boat into the harbor of Troy in the evening hours so that the city defenders would wait until morning to unload its contents. During the night, the men disembarked and set fire to the city [8].

Be that as it may, the metaphor of the Trojan Horse is common to this day to describe a strategy, most often, but not always malevolent, to introduce something or someone into a securely protected place. In that regard, CD71 serves a benevolent role in delivering antisense oligonucleotides to rescue differentiating erythroid progenitors [9]. Nonetheless, the molecular Trojan Horse role of CD71 manifests as it serves as the preferred port of endocytosis for human pathogenic hepatitis C virus [10], certain arenaviruses [11], the influenza A virus [12], the rabies lyssavirus [13], and the SARS-CoV2 whose Spike protein binds to CD71 with a high affinity (KD: ca. 2.95 nM) [14], and presumably others.

## Arenaviruses:

Viruses of the family Arenaviridae are generally, but not exclusively, spread by rodents, which serve as the virus' natural reservoir. They are characteristically pleomorphic, although they most often take on a spherical appearance with a diameter of 60-300 nm. Their envelope is covered with surface glycoprotein (GP) spikes that aid in their finding the binding receptor on the surface of the host cell. They possess a beaded nucleocapsid protein (NP) coat that encloses the viral RNA. They are typically bi- or tri-segmented ambisense single stranded (ss) RNA viruses with a complex genomic structure in that sections of their genome encode genes in the negative sense (i.e., reverse polarity) while other sections encode genes in the positive direction. They are nonetheless categorized as negative-sense ss RNA viruses. Typically, each RNA segment codes for two viral proteins in opposite orientation such that the negative-sense RNA genome serves as the template for transcription of a single mRNA and the positive-sense copy of the RNA genome templates for a second mRNA. The separate coding sequences of the two viral proteins are divided by an intergenic region RNA sequence that is predicted to fold into a stable hairpin structure. In this fashion, Arenavirus RNA segments are denoted Small (S), Medium (M, if present), and Large (L), and code for four viral proteins in a unique ambisense coding strategy. The S-segment (ca. 3.5 kb) encodes the viral nucleocapsid protein (NP) and glycoprotein (GC), whereas the L-segment RNA (ca. 7.2 kb) typically encodes the viral RNA-dependent RNA-polymerase (L) and the small RING-domain containing protein (Z). Protein Z must find GC and bind to it correctly in order to endow the virion the proper infectivity; Z must find and bind to L in order to activate the viral polymerase activity; and Z must find and associate with NP to promote virion packaging [15,16].

## Arenaviruses exist in at least four genera:

the Antennaviruses infect fish (i.e., frog fish, salmon); the Hartmaniviruses infect snakes as do the Reptarenaviruses; and the Mammarenaviruses infect mammals (i.e., rodents and human beings). The Mammarenavirus genus can cause serious and often lethal hemorrhagic fevers and inflammatory diseases, and consists of - thus far - 22 recognized virus species. It is divided into two serogroups based largely on geographical distribution, which diverged from a common ancestor 50- 45,000 years ago: "Old World" mammarenaviruses in the Eastern Hemisphere (i.e., Europe, Asia, Africa) associated with the Eurasian rodents in the family Muridae; and "New World" mammarenaviruses in the Western Hemisphere (i.e., North America, Central America, South America) associated with American rodents in the subfamily Sigmodontinae [17,18]. Only a few mammarenaviruses manifest ubiquitously worldwide, including the lymphocytic choriomeningitis (LCM) virus [15, 19-20,21, 22]. Old and New World mammarenaviruses are also distinct in terms of the IFN response they trigger in the host [23], and therefore, presumably in terms of the IFN-dependent processes of viral immunity and viral escape [24,25].

## Mammarenaviruses include, but are not limited to:

[1] New World - Machupo virus (MACV) that causes Bolivian hemorrhagic fever (BHF), also known as black typhus or Ordog Fever; Junín virus (JUN), responsible for Argentinian hemorrhagic fever, Sabiá virus (SABV), which indices Brazil hemorrhagic fever, Guanarito (GTOV), category A bioterrorism agent for its virulence as the etiological agent of Venezuelan hemorrhagic fever, and Whitewater Arroyo virus (WWAV) of the US Southwest, which leads to severe hemorrhagic fever with liver failure. They utilize the outermost apical ectodomain of CD71 as the point of attachment of their GP binding site for binding and penetration in the host cell [26- 27,28].

[2] Old World - Lassa virus (LASV), endemic to Africa, which causes flu-like symptoms that quickly degenerate into a massive inflammation syndrome leading to potentially lethal Lassa hemorrhagic fever; and Lymphocytic choriomeningitis (LCM), a rodent-borne viral infectious disease that begins with general malaise and headache and quickly progresses aseptic meningitis, encephalitis or serious meningoencephalitis. They use the laminin-binding member of the dystrophin glycoprotein complex, α-dystroglycan as their cellular receptor to enter target cell [27,29].

Alpha(α)-dystroglycan is an extracellular membrane glycoprotein that is anchored to the cell membrane via its transmembrane ligand, β-dystroglycan. Together, they associate into a component of the sarcolemmal dystrophin-glycoprotein complex, a specialized trans-membrane linker between the cytoskeleton (i.e., via the cytoskeleton protein dystrophin) and the extracellular matrix (i.e., via laminin). By mediating cytoskeleton-extra-cellular matrix communications, α-dystroglycan plays diverse and important roles in cell function, adhesion, migration, as well as serving as a receptor/co-receptor for bacterial and viral infection. Case in point, Old World mammarenaviruses utilize α-dystroglycan as their docking, binding site and port of entry through the plasma membrane [27,30,31].

## CD71 & CD81 synergy:

CD81 is the 26 kDa cell surface target of the anti-proliferative antibody (TAPA)-1 moiety, also known as Tetraspanin-28. It is involved in signal transduction and cell adhesion in the immune system: on B cells, CD81, as part of a complex with CD21, CD19, and Leu13, contributes to reducing the threshold for B cell activation via the B cell receptor by bridging Ag specific recognition and CD21-mediated complement recognition; on T cells, CD81 associates with CD4 and CD8 to provide a co-stimulatory signal to CD3/TcR [32]. CD71 is one of the several markers of activation of B and T lymphocytes, whose expression is, magnified several folds following stimulation via the B cell or the T cell receptor [33]. Moreover and as noted for CD71, CD81 is a co-receptor for certain viruses as well, from hepatitis C virus, to HIV-1and Chikungunya virus. In the case of hepatitis C virus infection, research has shown that anti-CD71antibody blocking lost its inhibitory activity after blocking to CD81, suggesting that CD71 may act synergistically as a post binding step, following, that is, binding of the virus to CD81 [10].

## Conclusion:

The role of CD71 in the infectivity of the arenaviruses is particularly important because they pose a threat to global public health owing to the emergence and re-emergence of highly fatal diseases (viz., acute febrile syndrome with coagulation abnormalities and generalized hemorrhage that may lead to life-threatening organ dysfunction [34]. It is likely and even probable that mammarenaviruses will increasingly play an important role in the context of climate change because as global warming inexorably continues to increase, and the Atlantic meridional overturning circulation (AMOC) continues to weaken, climate change will bring northward viruses and pathogens that were, until now, predominantly tropical, including the arenaviruses. Moreover, the thaw of eternal glacier ice and permafrost will extend releasing a myriad of ancient and novel viruses, bacteria and other pathogens [35]. Case in point, a novel arenavirus, named the Plateau Pika virus (PPV), which is capable of infecting mammalian cells, was recently obtained from the plateau pikas (Ochotona curzoniae) on the Qinghai-Tibet Plateau. Phylogenetic analyses revealed that PPV is a very ancient mammarenavirus that diverged from today's lines of New and Old World mammarenaviruses about 77-88 million years ago [36].

It is possible and even probable that PPV and other yet to be uncovered novel and ancient viruses emerging from permafrost thaw may also manifest affinities for CD71 receptor and for CD81 co-receptor. It follows that, whereas there is no anti-CD81 compound in clinical development at present, the current knowledge-base about the role of CD71 and CD81 in viral infections and immunopathology warrants more substantial considerations as a drug target [37].
